# Evolutionarily divergent DUF4465 domains have a common vitamin B_12_ ‐binding function

**DOI:** 10.1002/2211-5463.70231

**Published:** 2026-03-17

**Authors:** Charlea Clarke, Michal Banasik, Rokas Juodeikis, Martin J. Warren, Richard W. Pickersgill

**Affiliations:** ^1^ School of Biological and Behavioural Sciences Queen Mary University of London UK; ^2^ Quadram Institute Biosciences Norwich UK; ^3^ School of Natural Sciences University of Kent Canterbury UK

**Keywords:** B_12_‐binding proteins, B_12_‐scavenging proteins, DUF4465 (DUF Domain of Unknown Function), IPR027828 family proteins, microbiome, β‐jellyroll proteins

## Abstract

The DUF4465 family (DUF, domain of unknown function) contains more than 1000 members distributed across eight bacterial clades with species from diverse microenvironments including various gut microbiomes, hydrothermal vents, and soil. In the gut commensal *Bacteroides thetaiotaomicron* (*B. theta*), DUF4465 containing proteins act as high‐affinity B_12_–binding proteins that scavenge this cofactor to ensure bacterial survival. Such B_12_ capture is essential for bacteria that have lost the ability to synthesize B_12_ *de novo*. This raises the question of whether B_12_‐binding is ubiquitous across this family of proteins. Here, we show that B_12_‐binding is a recurrent function of eight distantly related members of the DUF4465 family. It is reasonable to conclude that B_12_‐binding is a common function of most DUF4465 proteins. These results establish DUF4465 as a structurally conserved family of augmented β‐jellyroll B_12_‐binding proteins with widespread roles in microbial competition for this essential cofactor.

**Impact statement**

DUF4465 defines a widespread, structurally conserved bacterial cobalamin‐binding domain and provides a promising scaffold for protein‐based B_12_ capture and purification.

## Graphical abstract

We show that DUF4465 family proteins, widespread across bacteria from gut microbiomes, hydrothermal vents, and soil, share a common vitamin B_12_‐binding function. These augmented β‐jellyroll proteins bind vitamin B_12_ via extended loops. Our findings establish sequence‐diverse DUF4465 proteins as a widespread class of B_12_‐binding proteins, highlighting their role in microbial competition for this essential micronutrient.

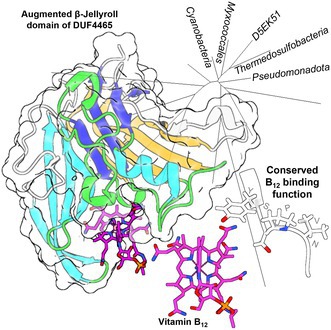

**Abbreviations**
B_12_
: vitamin B_12_/cobalamin
*B. theta*
: *Bacteroides thetaiotaomicron*
CCP4: collaborative computation project no. 4
DUF: domain of unknown function
EFI‐EST: enzyme function initiative enzyme similarity tool
ESRF: european synchrotron radiation facility
GNT: genome neighborhoodneighbourhood tool
IPR: interpro
IPTG: isopropyl β‐d‐1‐thiogalactopyranoside
MAFFT: multiple alignment using fast fourier transform
MP: maximum parsimony
MUSCLE: multiple sequence comparison by log‐expectation
NP: neighborneighbour‐joining
OD: optical density
PDB: protein data bank
PFAM: protein families database
RMSD: root mean square deviation
SSN: sequence similarity network
TCEP: tris(2‐CarboxyEthyl) phosphine

Nutrient acquisition is a critical aspect of bacterial survival, particularly for essential micronutrients, such as vitamin B_12_ (cobalamin or B_12_), which are often limited in the environment [1]. This scarcity is especially evident in the human gut, where only a minority of the bacterial species present can synthesize B_12_ completely [2, 3], and where over 80% of sequenced human microbial gut species nevertheless encode B_12_ dependent genes or riboswitches [4, 5]. In such ecosystems, microbial communities rely heavily on both sharing and competition for B_12_ [6, 7]. The importance of this cofactor stems from its central role in metabolism, acting as a cofactor for key enzymes such as methionine synthase and methyl malonyl CoA mutase [8, 9, 10]. Cobalamin biosynthesis is among the most complex and energetically demanding metabolic processes known, requiring approximately 30‐enzymatic step biosynthetic pathway [11, 12]. Although B_12_‐independent enzymes and pathways can substitute for survival, most bacteria that encode them also retain B_12_‐dependent isoenzymes and rely on transporters to acquire complete or incomplete corrinoids through salvage pathways [13, 14]. This pattern suggests that scavenging for B_12_ is not only sufficient but often preferred, as reflected in microbial communities where B_12_ producers are outnumbered by B_12_‐dependent organisms such as the gut commensal *B. theta* [15].

Recent studies have expanded our understanding of B_12_ transport and sequestration, particularly in B_12_‐limited environments such as the human gut. While the core B_12_ uptake pathway is highly conserved, scarcity exerts a strong selective pressure to diversify this protein network [16, 17, 18]. In *B. theta*, B_12_ limitation induces expression of additional outer membrane lipoproteins including those encoded by *btuG, btuH*, and *btuJ1/J2*, which are colocated within riboswitch‐regulated loci with essential transport machinery: *btuB, btuCD*, and *btuF*, *BtuG*. It has been shown that these lipoproteins bind B_12_ and enhance uptake efficiency, conferring a competitive edge under nutrient limitation [19, 20, 21]. Such adaptations highlight how otherwise conserved B_12_ transport systems evolve diversification in response to selective pressure. We recently demonstrated that the DUF4465 proteins BtuJ1 and BtuJ2 are high‐affinity B_12_‐binding proteins that localize to bacterial extracellular vesicles and promote the survival of *B. theta* [22]. Independent work has also reported B_12_‐binding to BtuJ1 aids bacterial survival [23]. Here, we show that B_12_ binding is a unifying feature of DUF4465 proteins, demonstrated across eight family members with as little as 16% sequence identity. Additionally, we show that these adaptations are not exclusive to gut bacteria, expanding our understanding of bacterial competition for micronutrients such as B_12_.

## Materials and methods

### Molecular biology

**Fig. 1:**
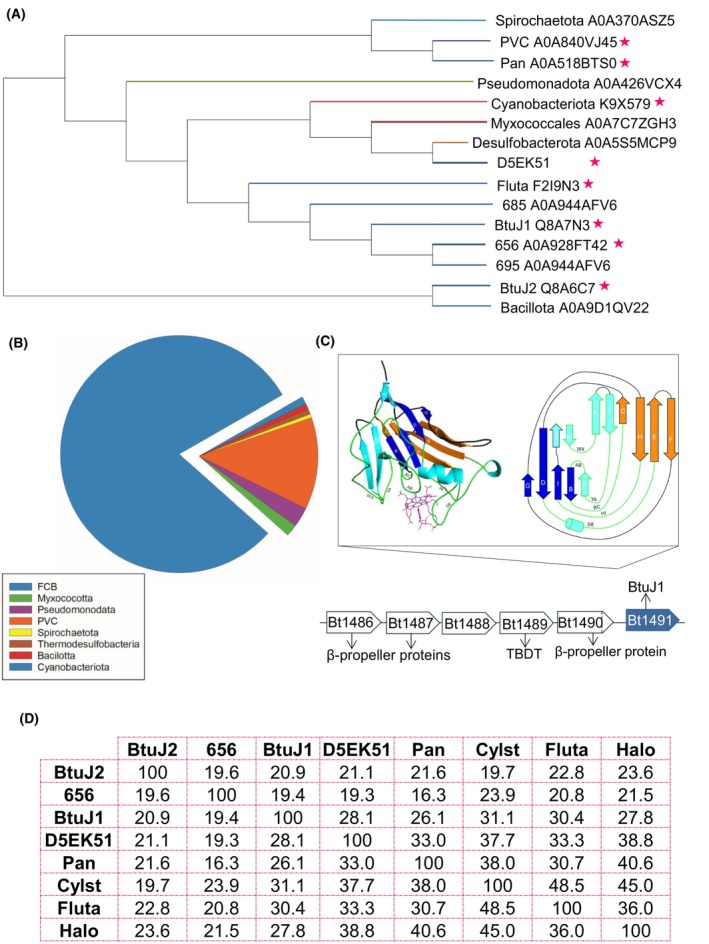
Evolutionary distribution, structural features, and genomic context of DUF4465 family proteins. (A) Phylogenetic tree of representative DUF4465 (IPR027828 family) protein sequences from diverse bacterial phyla. Branch lengths are proportional to sequence divergence. Pink stars indicate proteins selected for study. Sequences were aligned using MUSCLE5, and the tree was constructed using the MEGA11 software. (B) Taxonomic distribution of DUF4465 homologs shown as a proportional pie chart; the FCB group represents most sequences, with smaller contributions from *Myxococcota*, *Pseudomonadota*, PVC, *Spirochaetota*, *Thermodesulfobacteria*, *Bacillota*, and *Cyanobacteriota*. (C) To the left is shown the structure of *B. theta* DUF4465 protein (gene identifier *Bt1491*, recently designated *BtuJ1*) showing cartoon representation of the *β*‐jellyroll barrel with B_12_ in magenta lines (PDB code 9FCT), and right is the corresponding schematic *β*‐sheet topology diagram. This panel was produced using PyMol. The inset depicts the genomic neighborhood of *Bt1491*, showing its proximity to genes encoding *btuB*, *btuC*, *btuD*, and other predicted vitamin B_12_ transport proteins. (D) Pairwise sequence identity matrix for the DUF4465 proteins shown in panel A selected for assessment of B_12_‐binding function.

DNA corresponding to the soluble domains of the DUF4465 proteins, lacking the predicted lipoprotein motif, was inserted into a modified pET3a vector resulting in constructs encoding N‐terminal hexahistidine tagged proteins produced via the T7 promotor. The plasmids were validated by sequencing. The eight starred proteins in Fig. 1A were produced in this way.

### Protein expression

*E. coli* BL21(AI) cells from ThermoFisher™ were used for protein production. Transformants were selected using 100 μg·mL^−1^ ampicillin. Single colonies were used to inoculate 50 mL Luria broth (100 μg·mL^−1^ ampicillin) and cultured overnight at 37 °C with shaking. 10 mL of overnight culture was used to inoculate 800 mL fresh media and cultivated until the culture reached mid‐log phase, measured with an OD_600_ value of 0.6. At this point, the culture was cooled and induced with 0.5 mm Isopropyl β‐d‐1‐thiogalactopyranoside (IPTG). The induced culture was incubated at 20 °C overnight, while shaking. Bacteria were collected by centrifugation, and the obtained pellets kept at −20 °C until required.

### Protein purification

The bacterial pellet from 400 mL culture was resuspended in 100 mL of binding buffer 20 mm Tris‐Cl, 150 mm NaCl, 30 m imidazole, and 1 mm tris(2‐carboxyethyl) phosphine (TCEP), and sonicated on ice. The obtained cell lysates were clarified by ultracentrifugation at 100 000× ***g*** for 40 min at 4 °C. The resulting supernatant was applied to a HisTrap HP column containing 5 mL of chelating Sepharose. The His‐tagged DUF4465 protein was then eluted in 5 mL fractions with elution buffer (20 mm Tris‐Cl, 150 mm NaCl, 500 mm imidazole, and 1 mm TCEP). The eluted protein fractions were then concentrated and applied to a size‐exclusion chromatography column (Superde×200, 16/16) in buffer 20 mm Tris–HCl (pH 7.5), 150 mm NaCl, 1 mm TCEP. The fractions corresponding to the expected elution volumes for the purified DUF domains were collected, flash‐frozen in liquid nitrogen, and stored at −80 °C. To produce DUF proteins in complex with B_12_ excess cyanocobalamin was added to the cell lysate before the affinity column. Unbound B_12_ was removed during the HisTrap HP column washing step.

### Protein crystallization and structure determination

The D5EK51 protein in complex with B_12_ was concentrated to 20 mg·mL^−1^ (0.7 μm) for crystallization trials using sitting drop vapor diffusion trials set up a Mosquito Crystallization robot (TTP Labtech) using the commercial screens JCSG+, Structure 1 and 2, Morpheus, MembranePlus, Proplex, LMB, MIDAS+ (Molecular Dimensions and Hampton Research). Crystallization plates were incubated at 20 °C. Crystals of D5EK51 grew in MIDAS+ and Memgold2 commercial screens. Crystallization was achieved using the MIDAS+ condition 34–1, comprising 35% poly(acrylic acid sodium salt) 2100, 0.1 M HEPES (pH 7.5), and 0.2 M ammonium sulfate. Crystals were mounted and washed in cryoprotectant augmented with 20% glycerol and then flash‐frozen in liquid nitrogen in loops for data collection. Diffraction data were collected using the DECTRIS EIGER X 9 M at ESRF beamline BM30A. Structure determination, refinement, and validation used molecular replacement with an AlphaFold‐generated model of D5EK51 within the CCP4 suite [24, 25, 26]. All molecular graphics were prepared using PyMOL (The PyMOL Molecular Graphics System, Version 2.0 Schrödinger, LLC) [27].

### Sequence alignment and phylogenetic tree construction

Amino acid sequences of DUF4465 protein representatives D5EK51, Pan, Fluta, 656, Cylst, and Halo were collected from the UniProt database and aligned using MUSCLE5 [28]. The phylogeny was inferred using the Maximum Likelihood method and Le‐Gascuel model of amino acid substitutions and the tree with the highest log likelihood is shown [29]. The initial tree for the heuristic search was selected by choosing the tree with the superior log‐likelihood between a Neighbor‐Joining (NJ) tree and a Maximum Parsimony (MP) tree [30]. The NJ tree was generated using a matrix of pairwise distances computed using the p‐distance. The MP tree had the shortest length among 10 MP tree searches; each performed with a randomly generated starting tree. The evolutionary rate differences among sites were modeled using a discrete Gamma distribution across five categories (+G = 2.7645). The analytical procedure encompassed 15 amino acid sequences. The partial deletion option was applied to eliminate all positions with less than 95% site coverage resulting in a final data set comprising 169 positions. Evolutionary analyses were conducted in MEGA11 utilizing up to eight parallel computing threads [31].

### Sequence similarity network and genome neighborhood analysis

Multiple sequence alignments were obtained using MUSCLE5 and MAFFT v7 [28, 32, 33]. Proteins belonging to the IPR027828 family (DUF4465) were analyzed using the Enzyme Function Initiative Enzyme Similarity Tool (EFI‐EST) and Genome Neighborhood Tool (EFI‐GNT) (https://efi.igb.illinois.edu) [34, 35]. A Sequence Similarity Network (SSN) was generated by submitting the InterPro family identifier IPR027828, retrieving UniProtKB sequences clustered at 90% sequence identity to reduce redundancy. An alignment score threshold of 15 (corresponding to c. 40% sequence identity) was used to define edges, yielding two major clusters. For genomic context analysis, representative sequences from each connected component were submitted to EFI‐GNT, examining genes within ±10 open reading frames (ORFs). Gene neighborhoods were annotated based on EFI‐generated diagrams and UniProt functional descriptions. Analysis of the signal sequences was performed by SignalP 6.0 [36].

## Results

### Comparative analysis of the DUF4465 family

A preliminary phylogenetic tree constructed from the alignment using MEGA11 [31] illustrates the broad distribution and deep branching of DUF4465 homologs, Fig. 1A. The InterPro database [37] similarly shows that members of the DUF4465 family (IPR027828) are distributed across at least eight bacterial clades, indicating broad phylogenetic representation, Fig. 1B. To capture this diversity experimentally, representative proteins were selected to span the sequence space of the family: D5EK51 from *Coraliomargarita akajimensis*, Pan from *Mucisphaera* calidilacus (A0A518BTS0), Fluta from *Fluviicola taffensis* (F2I9N3), 656 from a *Rikenellaceae* bacterium (A0A928FT42), Cylst from *Cylindrospermum stagnale* PCC 7417 (K9X579), and Halo from *Haloferula luteola* (A0A840VJ45). All selected proteins contain predicted N‐terminal signal peptides according to SignalP [36, 38], consistent with localization to the periplasm or cell surface and supporting a potential role in extracellular or periplasmic B_12_ acquisition.

Within this family, the B_12_ scavenging protein from *B. theta* is encoded in B_12_ riboswitch‐regulated loci, structural analyses of BtuJ1 show a *β*‐jelly roll fold characteristic of DUF4465 proteins, Fig. 1C [22]. Notably, BtuJ1 has a close homolog in *B. theta*, BtuJ2, illustrating the presence of paralogous DUF4465 proteins within the same organism, a pattern commonly observed in nutrient scavenging systems and consistent with gene duplication within this family [23]. Initial pairwise comparisons showed that the previously characterized B_12_‐binding proteins BtuJ1 and BtuJ2 share relatively low overall sequence identity, suggesting substantial divergence within the family. To evaluate sequence conservation more systematically, DUF4465 sequences were retrieved from UniProt and aligned using MUSCLE5 [28, 39]. The resulting multiple sequence alignment revealed a wide range of sequence identity values (approximately 15–50%), Fig. 1D. Notably, substantial sequence variability was observed within the Bacteroidetes phylum, in some cases exceeding the divergence observed between different clades. For instance, protein 656 (A0A928FT42) from a *Rikenellaceae* bacterium exhibited a particularly low sequence identity compared to BtuJ1 despite belonging to the same broader phylogenetic grouping.

### Determination of B_12_ ‐binding to DUF4465 family proteins

To assess whether cobalamin binding is a ubiquitous function across the DUF4465 protein family, representative sequences were retrieved from the UniProt database [39]. Initially, several DUF4465 candidate sequences were analyzed and selected to maximize phylogenetic breadth and sequence divergence, ensuring coverage across major bacterial clades while avoiding over‐representation of closely related homologs. Signal peptide–truncated constructs were designed to promote soluble cytoplasmic expression, and six proteins (D5EK51, Pan, Fluta, 656, Cylst, and Halo) were taken forward based on successful recombinant expression.

Among these, D5EK51 from *Coraliomargarita akajimensis* was prioritized for structural studies because it reproducibly yielded diffraction‐quality crystals. All proteins were purified using a two‐step chromatographic workflow consisting of immobilized metal affinity chromatography followed by size‐exclusion chromatography. Purifications were performed under two parallel conditions: in the presence of excess B_12_ (cyanocobalamin) and in its absence, enabling direct comparison of ligand‐bound and apo states.

Evidence of B_12_ binding was apparent during purification. For D5EK51, Pan, Fluta, Halo, Cylst and 656, nickel affinity columns developed and retained a distinct pink coloration even after extensive washing when proteins were purified in the presence of B_12_, indicating stable retention of the protein‐B_12_ complex. Eluted fractions from these purifications were similarly pink, consistent with co‐elution of protein–B_12_ complexes. Protein containing fractions were confirmed by absorbance at 280 nm and SDS/PAGE analysis, while the presence of bound B_12_ was monitored via absorbance at 361 nm. Comparative UV–visible spectra of proteins purified in the presence and absence of B_12_ further supported cofactor association, Fig. S1. Proteins isolated with B_12_ displayed the characteristic cobalamin absorbance features, including a peak near 361 nm, which was absent in apo preparations. Notably, this peak exhibited a measurable shift relative to free B_12_ in solution, consistent with perturbation of the B_12_ environment upon protein binding. Such spectral changes are typical when cobalamin transitions from solvent‐exposed to a protein‐embedded state, where hydrogen‐bonding interactions, and local polarity alter the ligand's electronic environment These data therefore provide spectroscopic evidence that B_12_ is not merely copurifying nonspecifically but is engaged in a defined binding environment within DUF4465 proteins.

**Fig. 2:**
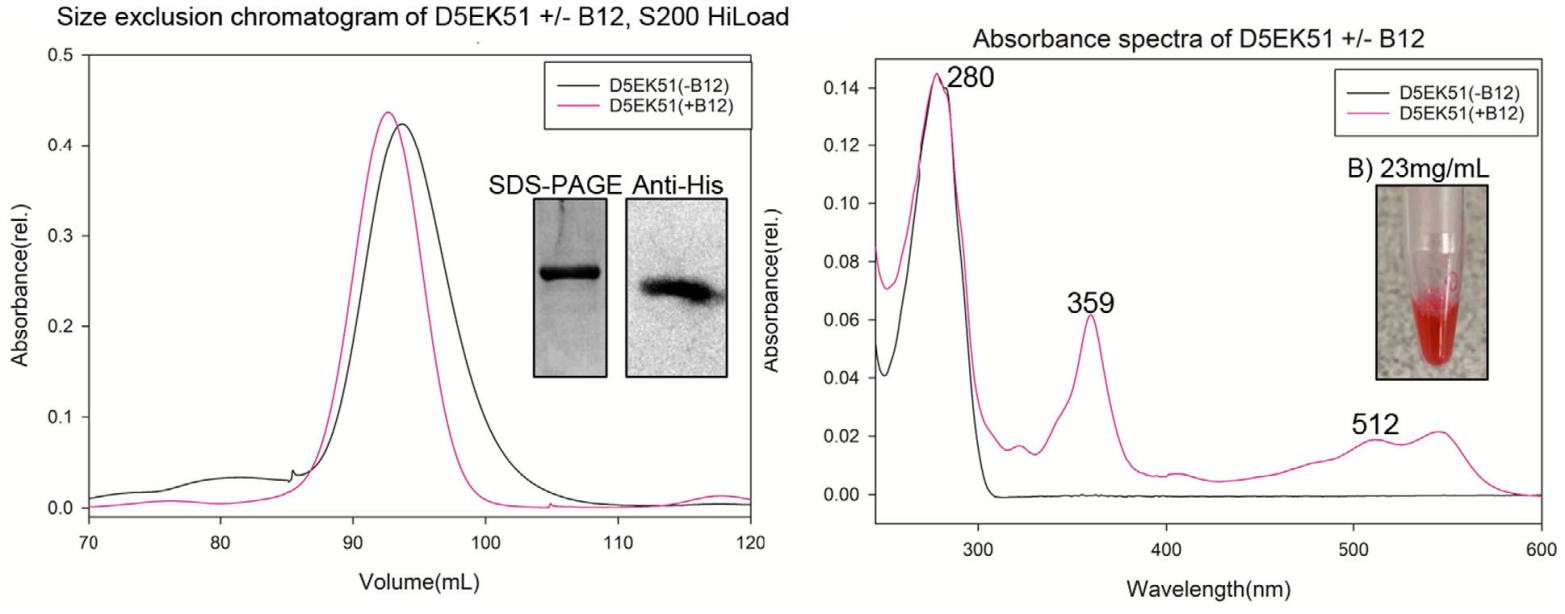
Spectroscopic and chromatographic characterization of the D5EK51–B_12_ complex. (A) Size‐exclusion chromatograms of D5EK51 purified with (pink line) or without (black line) B_12_ on a Superdex 200 HiLoad column. Both samples elute at ~95 mL, corresponding to the monomeric form of D5EK51 (26.9 kDa). SDS/PAGE and anti‐His western blot (inset) confirm the purity and presence of D5EK51 in both conditions. (B) UV–visible absorbance spectra of purified D5EK51 alone (black) and in complex with cyanocobalamin (pink). The D5EK51–B_12_ complex exhibits distinct absorbance features at 361 nm and 550 nm, consistent with B_12_ binding, while the unbound protein shows only the characteristic 280 nm absorbance. The inset shows the deep pink coloration of the D5EK51–B_12_ complex at 23 mg·mL^−1^, indicative of bound cobalt‐containing cobalamin.

Size exclusion chromatography profiles also revealed clear ligand‐dependent differences in elution behavior. Chromatograms of the D5EK51–B_12_ complex showed a reproducible leftward shift in elution volume relative to the apo protein, Fig. 2. The peak is also narrower indicating a more ordered state consistent with the ordering of the flexible loops upon ligand‐binding. Similar trends were observed for the other B_12_‐binding homologs, supporting the conclusion that cobalamin binding is a conserved biochemical feature within these DUF4465 proteins.

### Determination of the structure of the D5EK51‐B_12_ complex

The D5EK51‐B_12_ complex was successfully purified and concentrated, facilitating the growth of protein crystals. The resulting crystals belonged to the space group P3_2_21, with three D5EK51‐B_12_ complexes in the asymmetric unit. Structural alignment of chains 2 and 3 to chain 1 yielded root‐mean‐square deviations (RMSDs) of 0.154 Å and 0.084 Å, respectively, over 170 equivalent Cα atoms, indicating high structural similarity. Structure determination was performed using molecular replacement with an AlphaFold‐generated [24, 25] model of D5EK51 within the CCP4 suite [26]. The structure was refined to a resolution of 1.85 Å, achieving R_work_ and R_free_ values of 0.198 and 0.222, respectively. Notably, the final model exhibited no Ramachandran outliers, underscoring its stereochemical quality. Crystallographic and refinement statistics are summarized in Table S1.

### Structural features and B_12_ binding mechanism

**Fig. 3:**
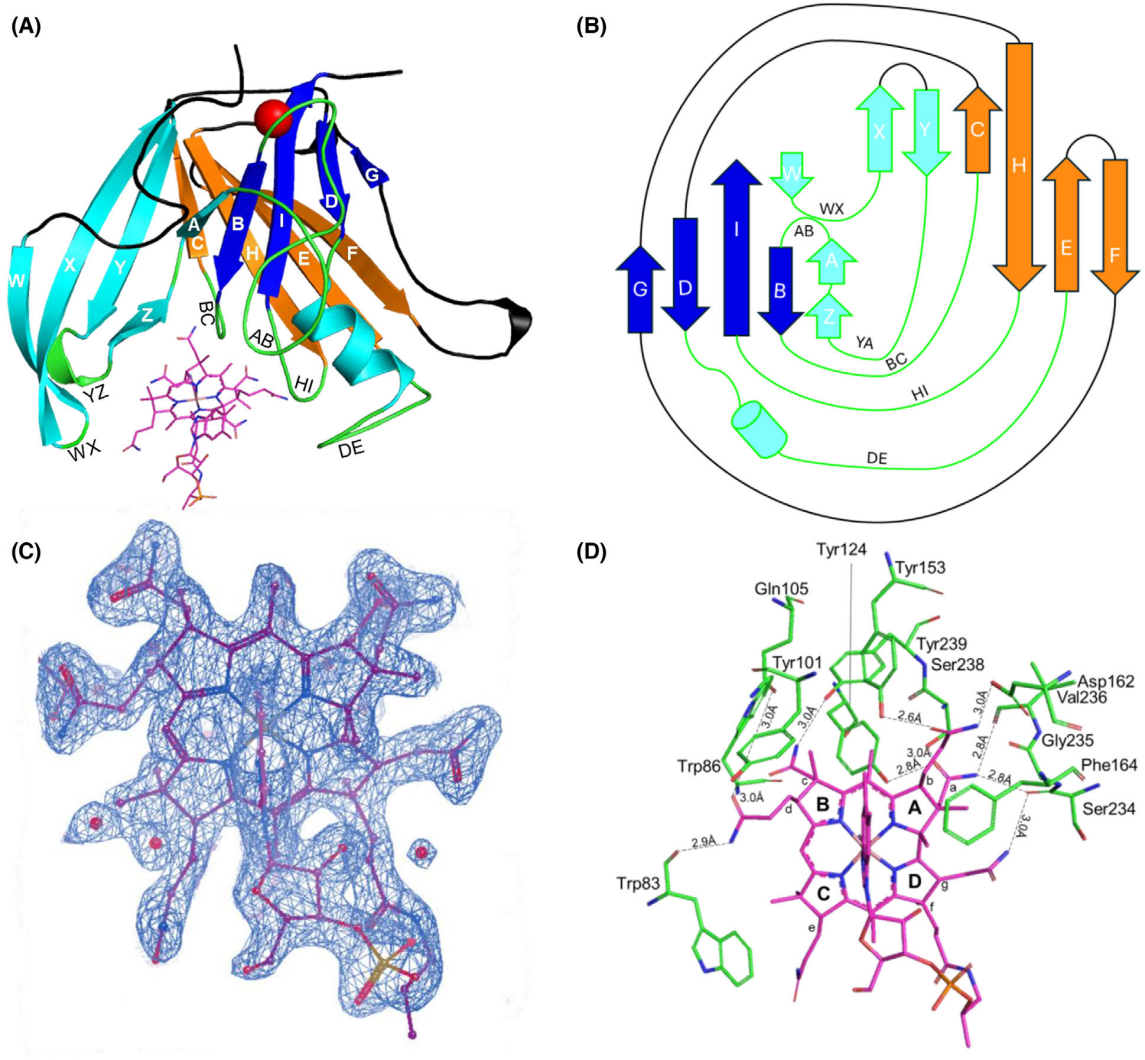
Structural features of the D5EK51–B_12_ complex. (A) Overall cartoon representation of the D5EK51 structure, highlighting the augmented *β*‐jellyroll fold characteristic of DUF4465 family proteins. The two *β*‐sheets are labeled according to convention: BIDG (blue) and CHEF (orange). Five additional *β*‐strands (WXYZA) at the N terminus and a loop‐associated *α*‐helix (loop DE) are also present. Loops are named according to the *β*‐strands they link together; hence loop HI connects *β*‐strands H and I. Loops forming the B_12_‐binding site are shown in green. Cyanocobalamin is depicted in magenta, and a stabilizing calcium ion is shown as a red sphere. (B) Topology diagram of the D5EK51 secondary structure. The *β*‐jellyroll fold consists of two antiparallel four‐stranded *β*‐sheets with additional N‐terminal *β*‐strands (WXYZA) and an α‐helix (loop DE), consistent with other DUF4465 family proteins. (C) 2Fo–Fc electron density map (blue mesh) of the B_12_ ligand contoured at 1.0*σ*, showing well‐resolved density for the corrin ring and side chains. Ordered water molecules are also visible and contribute to ligand binding. (D) Close‐up view of the B_12_‐binding pocket showing the hydrogen bonding network (dashed lines). Eleven hydrogen bonds are formed between D5EK51 and B_12_, involving both main‐chain and side‐chain atoms. These interactions predominantly occur near the A‐ring of the corrin ring, stabilizing B_12_ within a hydrophobic cavity composed of aromatic residues shown in green. This Figure was produced using PyMol.

The D5EK51 protein adopts an augmented β‐jellyroll fold. This fold consists of eight β‐strands arranged into two antiparallel four‐stranded β‐sheets, BIDG and CHEF, as shown in Fig. 3A,B. In D5EK51, the β‐jellyroll architecture is extended by five additional antiparallel β‐strands at the N terminus (WXYZA) and an additional α‐helix inserted within loop DE. This structural arrangement is consistent with other recently solved DUF4465 family members, BtuJ1 and BtuJ2 (PDB codes: 9FCT and 9I2L [22]).

Cobalamin is well‐resolved within each D5EK51 molecule of the asymmetric unit, Fig. 3C. The density for the upper ligand of cyanocobalamin is not as strong as for the rest of the molecule, which is evidence of X‐ray‐induced reduction of the cyanocobalamin's cobalt centre to give cob(II)alamin [40]. An upper limit to the *K*_d_ for cobalamin can be determined from the crystallization conditions as the cobalamin site is fully occupied in the electron density map. As the protein and ligand concentrations are 0.7 μm, *K*_d_ will be less than 0.2 μm and is likely much lower than this value but possibly not as low as the *K*_d_ of BtuJ1 measured at circa 10^−10^ M. The cobalamin binding pocket is formed by six loops conserved among DUF4465 proteins and is primarily lined with aromatic hydrophobic residues, including tyrosine, phenylalanine, and tryptophan. These aromatic residues not only accommodate the corrin ring but also form contacts with its side chains, facilitating tight binding, Fig. 3D.

In addition, a network of 11 hydrogen bonds is formed between D5EK51 and the side chains of B_12_, involving both backbone and side‐chain protein atoms. This dense hydrogen‐bonding network is comparable to those observed in the well‐characterized cobalamin‐binding proteins BtuJ1 and BtuJ2, which form 16 and 13 hydrogen bonds with the B_12_, respectively. PDBePISA analysis further defines the contribution of nonpolar interactions to complex stability, highlighting extensive hydrophobic surface complementarity between the protein and the corrin ring. All crystallized DUF4465–B_12_ complexes show substantial ligand burial, with more than 50% of the B_12_ solvent‐accessible surface area occluded upon binding. For D5EK51, the buried surface area is 688Å^2^ (56%), compared with 775Å^2^ (62%) for BtuJ1 and 857Å^2^ (69%) for BtuJ2 [41]. The magnitude of surface burial and multiple polar interactions together indicate a tightly packed, specifically coordinated binding interface. D5EK51 shares 28% and 21% sequence identity with BtuJ1 and BtuJ2 and their structures align with RMSD of 3.548 Å and 1.518 Å, respectively. The HI loop and the DE loop/helix (named after the β‐strands connected) are highly conserved among the three structures, both in their conformation and in the identity of B_12_‐coordinating residues. These loops contain the conserved B_12_‐binding tyrosine's, on loop HI (Y245 in BtuJ1 numbering) and within the *α*‐helix of loop DE (Y146), highlighting a preserved functional core of the DUF4465 family.

**Fig. 4:**
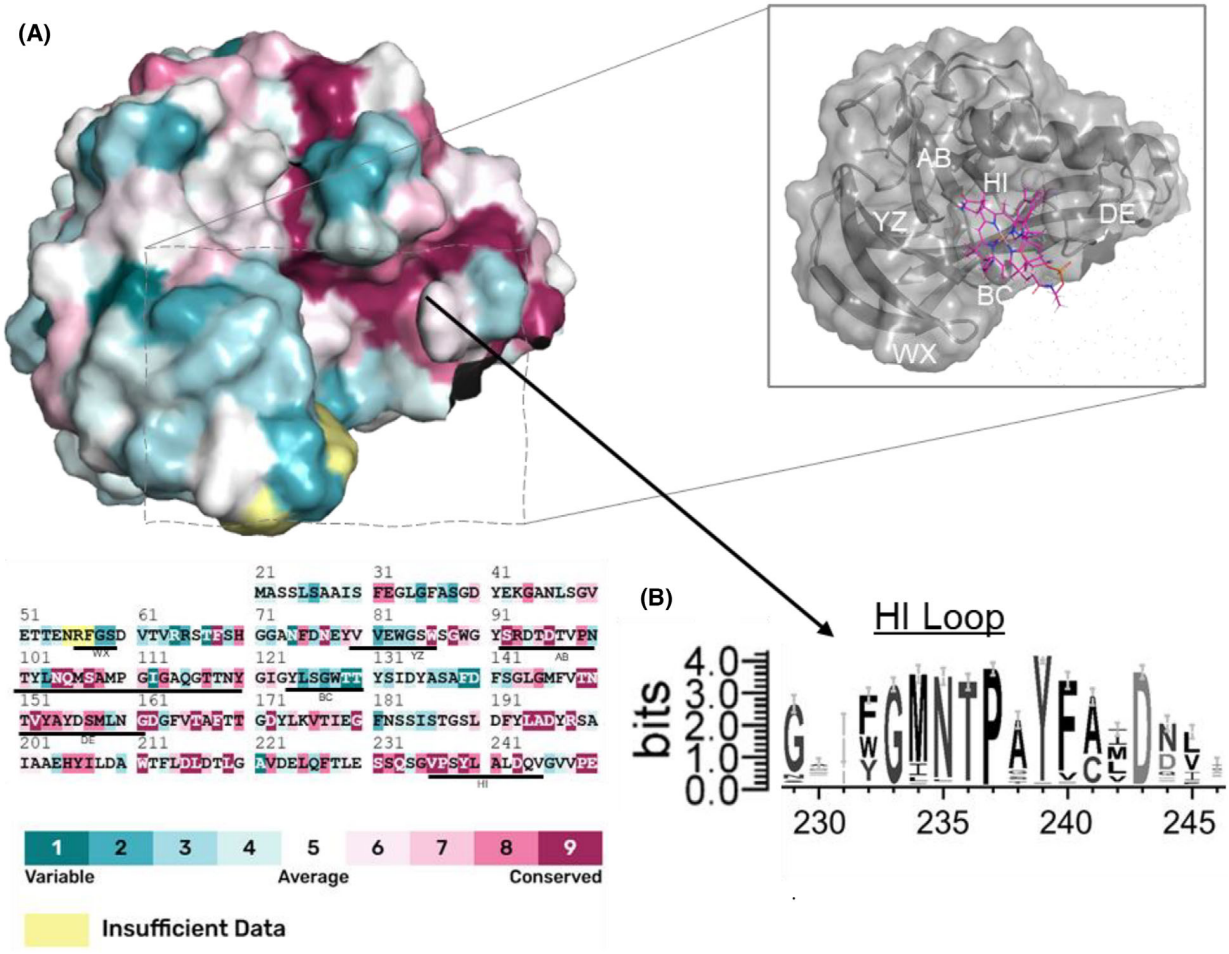
ConSurf analysis of D5EK51 reveals evolutionary conservation of the B_12_‐binding pocket. (A) Surface representation of the experimentally determined structure colored by residue conservation (teal indicates variable and pink conserved residues), highlighting the conserved B_12_‐binding pocket (the boxed region indicates the positions of the loops). Below is a multiple sequence alignment of representative homologs with conservation scores (1–9) mapped to the structure. The surface conservation and corresponding sequence alignment shown in this Figure was generated using the ConSurf server. (B) Sequence logo for residues predicted on HI loop, showing a conserved NTPAY motif.

Surface conservation mapping further supports this conclusion, Fig. 4A. Key aromatic residues within the binding pocket are conserved across homologs, particularly those in loops YZ, AB, BC, HI, and DE. The HI loop (GTPAYF) is especially conserved, with all residues present at greater than 70% frequency and the critical B_12_‐binding tyrosine retained in 97% of homologs [42]. Sequence motif alignment was used to create a sequence logo, Fig. 4B and extension of this analysis representatives of all eight clades strongly support B_12_ binding as a conserved function across the DUF4465 family.

### Genomic analysis of genetic context of DUF4465 encoding genes

We identified two major genomic clusters of DUF4465 proteins [34, 35]. The first, comprising approximately 900 members, showed frequent colocalization with transport‐associated domains: 20% colocated with ABC‐type transporters (PF00005), 40% with TonB‐dependent β‐barrels (PF00593), 55% with their associated plug domains (PF07715), and 40% with β‐propeller domain proteins (PF16819). The second cluster, containing 195 DUF4465 members, exhibited even stronger associations, with 80–95% co‐occurring with these transport systems and periplasmic binding proteins. In total, 84 DUF4465 proteins lacked neighboring Pfam‐annotations, suggesting that these values may underestimate the true frequency of such associations. Together, these patterns indicate that DUF4465 proteins are frequently genomically linked to vitamin B_12_ transport systems, supporting their role in B_12_ acquisition.

## Discussion

DUF4465 proteins provide valuable insight into how bacteria handle the transport of a complex and fragile cofactor such as vitamin B_12_. Among the suite of B_12_‐handling proteins, there is strong selective pressure to evolve architectures that tightly bind the cofactor; both because B_12_ is scarce in most environments and because it is chemically unstable. Vitamin B_12_ is well known to be sensitive to light, heat, oxidation, and pH, all of which can lead to rapid degradation under many physiological conditions [43, 44]. It is therefore unsurprising that such a vital cofactor is almost universally trafficked via dedicated protein chaperones that protect it from damage and ensure its safe delivery into the cell for enzymatic use [45, 46].

Previous analyses of B_12_‐bound structures in the Protein Data Bank (PDB) have identified similar patterns described here, such as a ‘hydrophobic shroud’ in the B_12_ binding pocket [47]. However, many of these structures correspond to enzymes, which are designed to retain B_12_ as a cofactor rather than transfer it to other proteins. In contrast, our analysis focuses on B_12_‐binding proteins involved in scavenging and transport, providing new insight into a distinct class of B_12_ interaction mechanisms.

**Fig. 5:**
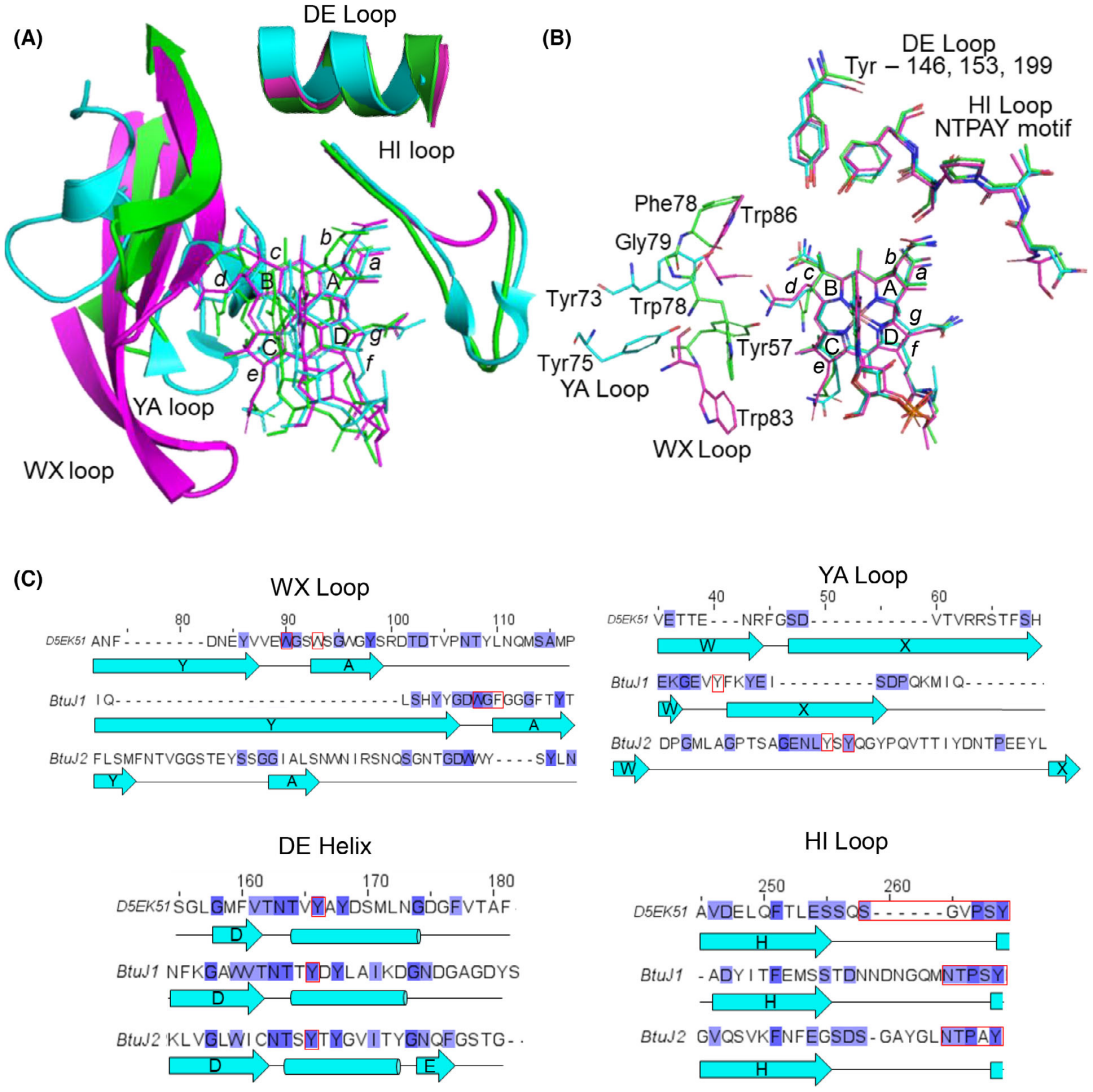
Comparison of the B_12_‐binding loops of D5EK51, BtuJ1 and BtuJ2. (A) Cartoon representation of the HI, DE, WX and YA B_12_‐binding loops of D5EK51 (magenta), BtuJ1 (green) and BtuJ2 (cyan) together with their bound B_12_ molecules after superimposition (PDB codes: 9HMU, 9FCT, and 9I2L respectively). The N‐terminal WX and YA loops vary significantly, but the C‐terminal DE and HI loops are highly conserved. (B) Close‐up view of the B_12_ binding site reveals minimal conservation of aromatic and hydrogen bonding residues responsible for ligand coordination on the YA, WX side of the pocket. However, side chains from all three structures show near‐identical orientation around the A ring of the B_12_ molecule (DE and HI loops), highlighting a shared ligand recognition strategy. (C) Structure‐based sequence alignment of the loops from BtuJ1, BtuJ2, and D5EK51. Conserved residues involved in B_12_‐binding are boxed in red, and secondary structure elements are shown as arrows (*β*‐strands) and cylinders (*α*‐helices). This Figure was produced using PyMol.

The DUF4465 proteins use a common binding mechanism involving loops that provide shape complementarity and a high density of hydrophobic residues, Fig. 5. Notably, loops are also often seen binding B_12_ in other bacterial B_12_ transport proteins, BtuK, BtuH, BtuG, BtuB, BtuF.

Therefore, a loop pocket architecture, supported by the conserved β‐jellyroll scaffold in DUF4465 proteins, appears to be the solution for achieving high affinity and conditional release. The extensive hydrogen‐bonding network from the loops consistently observed in DUF4465 and other B_12_ transport proteins further stabilizes the cofactor while still allowing partial solvent exposure; this is a structural signature of proteins specialized for transfer rather than catalysis. Interestingly, in eukaryotic B_12_ transport proteins, the B_12_ is encapsulated by two domains, which shield it from solvent; these proteins do not perform transfer and are instead internalized by the cell and digested [48].

Despite consistently conserved features in DUF4465 proteins, detailed analysis of the D5EK51 crystal structure and sequence reveals differences compared to BtuJ1 and BtuJ2, particularly in the N‐terminal region of the DUF4465 fold. One notable variation is the auxiliary *β*‐strands at the N terminus (*β*‐strands W, X, and Y, Fig. 3). While the number of these strands varies among family members, BtuJ1 and BtuJ2 each possess four auxiliary strands, whereas D5EK51 contains five (W, X, Y, and Z, A, Fig. 3). This seemingly minor difference has a significant impact on the topology of the binding pocket, especially the lengths and conformations of loops WX and YA. Overall, the WX and YA loop architecture varies markedly across available structures and shows lowest conservation; a sequence alignment is shown in Fig. S2. BtuJ2 exhibits considerably longer WX/YA loops, whereas both BtuJ1 and D5EK51 have shorter loops. These loops, along with the auxiliary strands, shape the N‐terminal side of the pocket (left‐side as shown in Fig. 5) responsible for coordinating the B_12_ B and C rings; they may also be critical for lower ligand recognition or for protein–protein transfer interactions due to their position. Additionally, we observe distinct hydrogen‐bonding patterns emerging at the N‐terminal end of the binding site for different DUF4465 proteins. BtuJ1 employs a conserved tryptophan residue to π‐stack against the adenosyl lower ligand of the B_12_ molecule. In contrast, BtuJ2/D5EK51 uses two tyrosine/tryptophan residues to achieve a similar anchoring function but via direct hydrogen bonding, avoiding contact with the lower ligand. While both mechanisms effectively coordinate side chains from C and D of the B_12_ corrin ring, they result in slightly different ligand conformations, specifically of the *d* B_12_ side chain.

Further identification of DUF4465 family members and the biochemical confirmation of their tight B_12_‐binding expands our understanding of their role in bacterial B_12_ scavenging. Particularly, the widespread distribution of DUF4465 homologs across diverse bacterial lineages suggests that this mode of B_12_ handling is not confined to gut‐associated species but rather represents a general adaptation to the scarcity of B_12_ in most environments. This finding challenges the prevailing view that related dedicated B_12_ scavenging systems are a feature unique to *B. theta* and related gut microbes. Instead, the presence of DUF4465 proteins across multiple phyla suggests that bacteria from varied ecological niches have adopted similar strategies for capturing and stabilizing cobamides.

Genomic analyses further support this conclusion: DUF4465‐encoding genes are frequently co‐located with known B_12_ transporter loci, implying a coordinated role in uptake and trafficking. Notably, over 70% of these proteins are predicted lipoproteins, with the remainder bearing periplasmic targeting signals; a pattern consistent with localization to the outer membrane and function at the cell–environment interface. In addition, DUF4465 proteins are often genomically associated with other “DUF” protein families that share structural similarity with accessory B_12_‐scavenging proteins from *B. theta*. But due to naming convention inconsistencies, it is more difficult to group these proteins together.

These results suggest that while not all bacteria may invest in such specialized machinery, a substantial proportion do, likely reflecting ecological or metabolic trade‐offs in cobamide acquisition. Interestingly, this same selective pressure is mirrored in laboratory evolution studies; Mok et al.[49] showed that *E. coli*, when forced to grow with a suboptimal B_12_ analog, rapidly accumulates mutations that enhance B_12_ transport and activation [49]. Although such experiments cannot fully capture the complexity of natural ecosystems, they reinforce the idea that strengthening cobamide uptake and handling is a universal adaptive solution to cofactor limitation.

## Conflict of interest

The authors declare no conflict of interest.

## Author contributions

CC was involved in conceptualization, methodology, formal analysis, investigation, writing – original draft, writing – review and editing. MB was involved in methodology, formal analysis. RJ was involved in methodology. MW was involved in conceptualization, funding acquisition. RWP was involved in conceptualization, writing – original draft, writing – review and editing, supervision, funding acquisition.

## Supporting information


**Table S1.** Crystallographic and refinement statistics for D5EK51 (PDB code: 9HMU).
**Fig. S1.** Spectroscopic and chromatographic characterization of the Pan, Halo, 656, Fluta, Cylst–B_12_ complexes.
**Fig. S2.** Sequence alignment showing conservation and the positions of secondary structures of D5EK51, BtuJ1 and BtuJ2.

## Data Availability

The structure and structure factors for DUF4465 protein D5EK51 are deposited in the protein databank (PDB) with code 9HMU.
