# Supplementary material for: Evolutionarily divergent DUF4465 domains have a common vitamin B12 ‐binding function
Source: FEBS Open Bio. 2026 Mar 17;16(8):1537–49. doi: 10.1002/2211-5463.70231 (PMC13398666; doi:10.1002/2211-5463.70231)

**Supplementary Table 1.** Crystallographic and refinement statistics for D5EK51


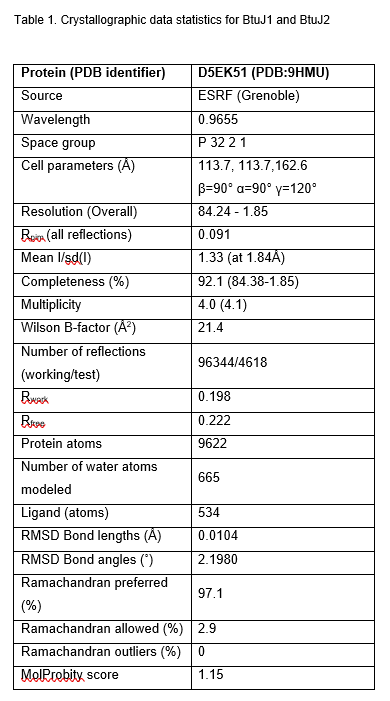


**Supplementary Figure 1**. Spectroscopic and chromatographic characterization of the Pan, Halo, 656, Fluta, Cylst–B_12_ complexes. Each protein has a panel which shows the absorption spectra of the protein eluted from the affinity column. The spectra show characteristic B_12_ absorption peaks. The inset confirms the presence of the purified protein at the expected molecular mass using Western blot and the pink colour of the eluted protein in the curvette clearly shows that the protein binds B_12_. The details for D5EK51 are presented in the main text.


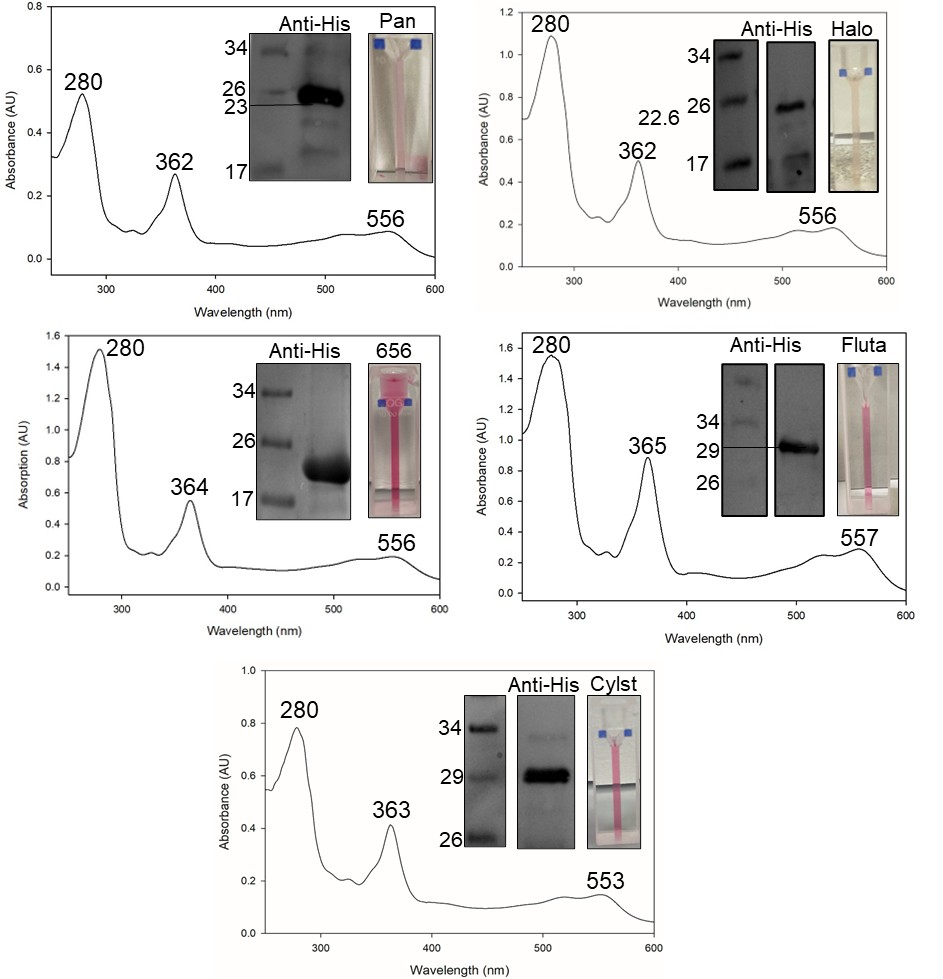


**Supplementary Figure 2. Sequence alignment of D5EK51 and BtuJ1 and BtuJ2.** The full structure-based sequence alignment of BtuJ1, BtuJ2, and D5EK51, with secondary structure elements annotated. Conserved residues involved in B_12_ binding are indicated inside red boxes, and secondary structure elements are shown as arrows (β-strands) and cylinders (α-helices). Sequences were aligned using MUSCLE5, the resulting alignment is displayed using Jalview software.


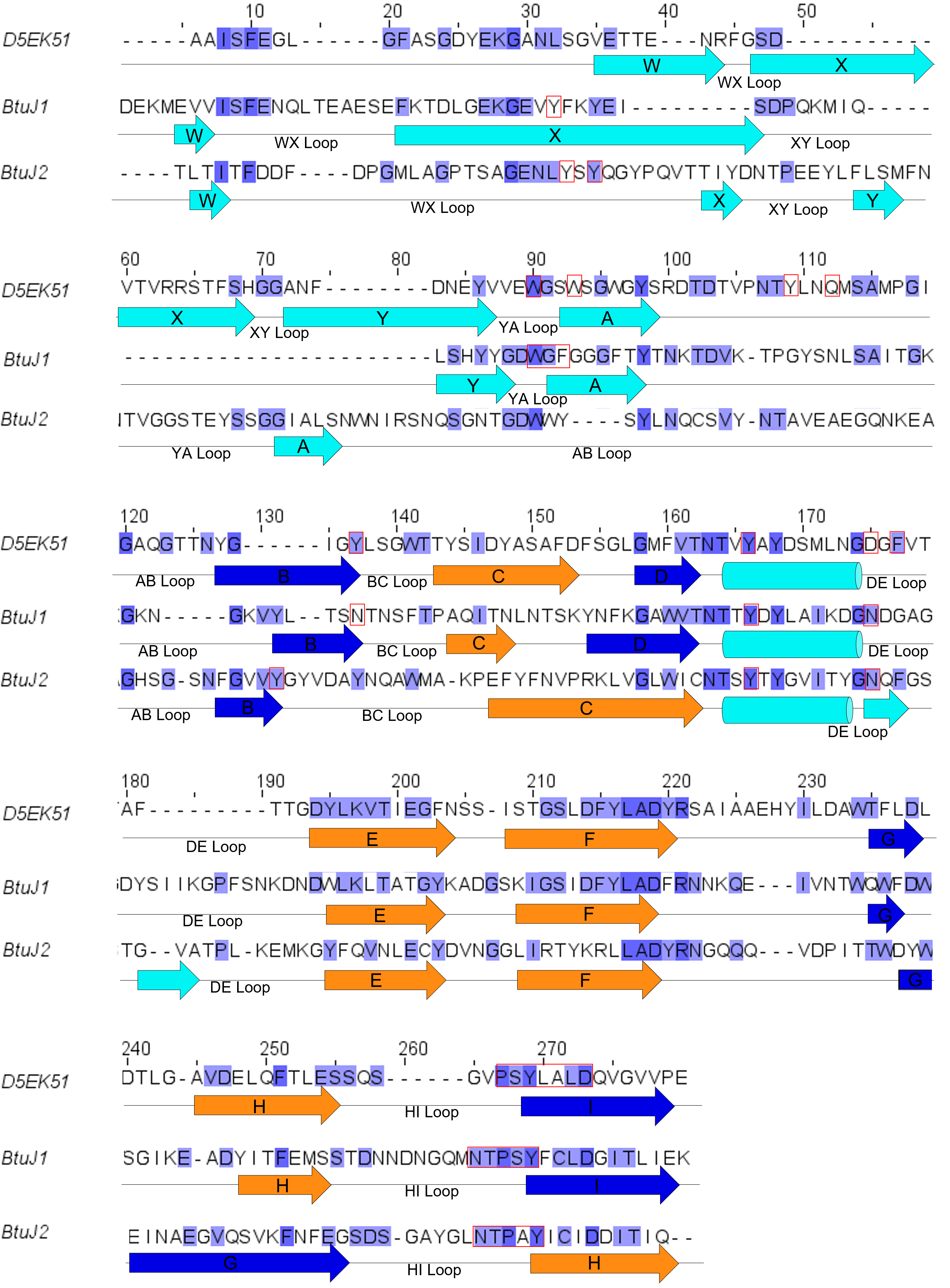

Supplement: Supplementary file 1 — Table S1. Crystallographic and refinement statistics for D5EK51 (PDB code: 9HMU). Fig. S1. Spectroscopic and chromatographic characterization of the Pan, Halo, 656, Fluta, Cylst–B12 complexes. Fig. S2. Sequence alignment showing conservation and the positions of secondary structures of D5EK51, BtuJ1 and BtuJ2. [file FEB4-16-1537-s001.docx]
